# Development of a PCR-Based Reverse Genetics System for an Attenuated Duck Tembusu Virus Strain

**DOI:** 10.1371/journal.pone.0156579

**Published:** 2016-06-01

**Authors:** Xiaogang Wu, Ying Shi, Dawei Yan, Xuesong Li, Pixi Yan, Xuyuan Gao, Yuee Zhang, Lei Yu, Chaochao Ren, Guoxin Li, Liping Yan, Qiaoyang Teng, Zejun Li

**Affiliations:** Department of Avian Infectious Disease, and Innovation Team for Pathogen Ecology Research on Animal Influenza Virus, Shanghai Veterinary Research Institute, Chinese Academy of Agricultural Science, Shanghai, China; Sun Yat-sen University, CHINA

## Abstract

The infectious disease caused by the duck Tembusu virus (DTMUV) has resulted in massive economic losses to the Chinese duck industry in China since 2010. Research on the molecular basis of DTMUV pathogenicity has been hampered by the lack of a reliable reverse genetics system for this virus. Here we developed a PCR-based reverse genetics system with high fidelity for the attenuated DTMUV strain FX2010-180P. The rescued virus was characterized by using both indirect immunofluorescence assays (IFA) and whole genome sequencing. The rescued virus (rFX2010-180P) grew to similar titers as compared with the wild-type virus in DF-1 cells, and had similar replication and immunogenicity properties in ducks. To determine whether exogenous proteins could be expressed from DTMUV, both an internal ribosomal entry site (IRES) and the enhanced green fluorescent protein (eGFP) gene were introduced between the NS5 gene and the 3' non-coding sequence of FX2010-180P. A recombinant DTMUV expressing eGFP was rescued, but eGFP expression was unstable after 4 passages in DF-1 cells due to a deletion of 1,294 nucleotides. The establishment of a reliable reverse genetics system for FX2010-180P provides a foundation for future studies of DTMUV.

## Introduction

The newly emerged duck infectious disease caused by duck Tembusu virus (DTMUV), family Flaviviridae, genus Flavivirus, has resulted in massive losses to the duck industry in China since 2010[1, 2]. Tembusu viruses have spread widely throughout mainland China and have been isolated from ducks, geese, chickens, pigeons, sparrows, and mosquitos, suggesting this virus has a wide host range[3–9]. A majority of sampled duck industry workers in Shandong Province (71.9% of 132 serum samples) had antibodies against DTMUV and 47.7% of oral swabs were positive for viral RNA, indicating the possibility of human infections[10]. It is thus urgent to study DTMUV virulence mechanisms but the lack of a reliable reverse genetics system has hampered these studies.

Viral reverse genetic systems have been developed successfully for many other flaviviruses, including Yellow fever virus[11], Dengue virus[12, 13], West Nile virus[14], Japanese encephalitis virus[15, 16], Tick-borne Langat virus[17], and Omsk hemorrhagic fever virus[18]. However, these systems are difficult to develop because flavivirus genomes tend to be unstable in bacterial hosts during cDNA cloning [19–24]. Indeed, an infectious full-length DTMUV cDNA clone was extensively mutated in bacterial hosts [25]. The PCR-based reverse genetic systems established for Japanese Encephalitis virus, Dengue virus, and West Nile virus [12, 26–29] have however suggested new experimental strategies.

A live, attenuated DTMUV vaccine candidate strain, FX2010-180P, was recently developed through serial passaging of FX2010 through chicken embryo fibroblasts (CEFs) [30]. FX2010-180P had no pathogenicity in ducks, but protected ducks from infection by wild-type DTMUV. The attenuation of FX2010-180P was due to 19 amino acid changes and 15 synonymous mutations. The DTMUV genome contains 10,991 nucleotides with an open reading frame (ORF) of 10,278 nucleotides flanked by 5' and 3'-non-coding regions (NCRs) of 95 and 618 nucleotides, respectively[31–35]. Here we developed a reverse genetics system for FX2010-180P using a high-fidelity DNA polymerase and characterized the rescued virus. A virus expressing eGFP was rescued using this system to explore the possibility of the attenuated DTMUV as a viral vector.

## Materials and Methods

### Cells and virus

DF-1 cells were obtained from the American Type Culture Collection and maintained in Dulbecco's Modified Eagle Medium (DMEM), supplemented with 10% fetal bovine serum (FBS). The duck Tembusu Virus FX2010-180P strain was used for viral RNA extraction.

### Animals

Ducks were hatched from SPF Shelduck eggs (Harbin Veterinary Research Institute) and housed in isolators for 4 to 6 weeks for experiments. The Animal Care and Use Committee of the Shanghai Veterinary Research Institute, Chinese Academy of Agricultural Sciences approved the animal experiments.

### Construction of recombinant plasmids containing fragmented viral genome sequence

**Fig 1:**
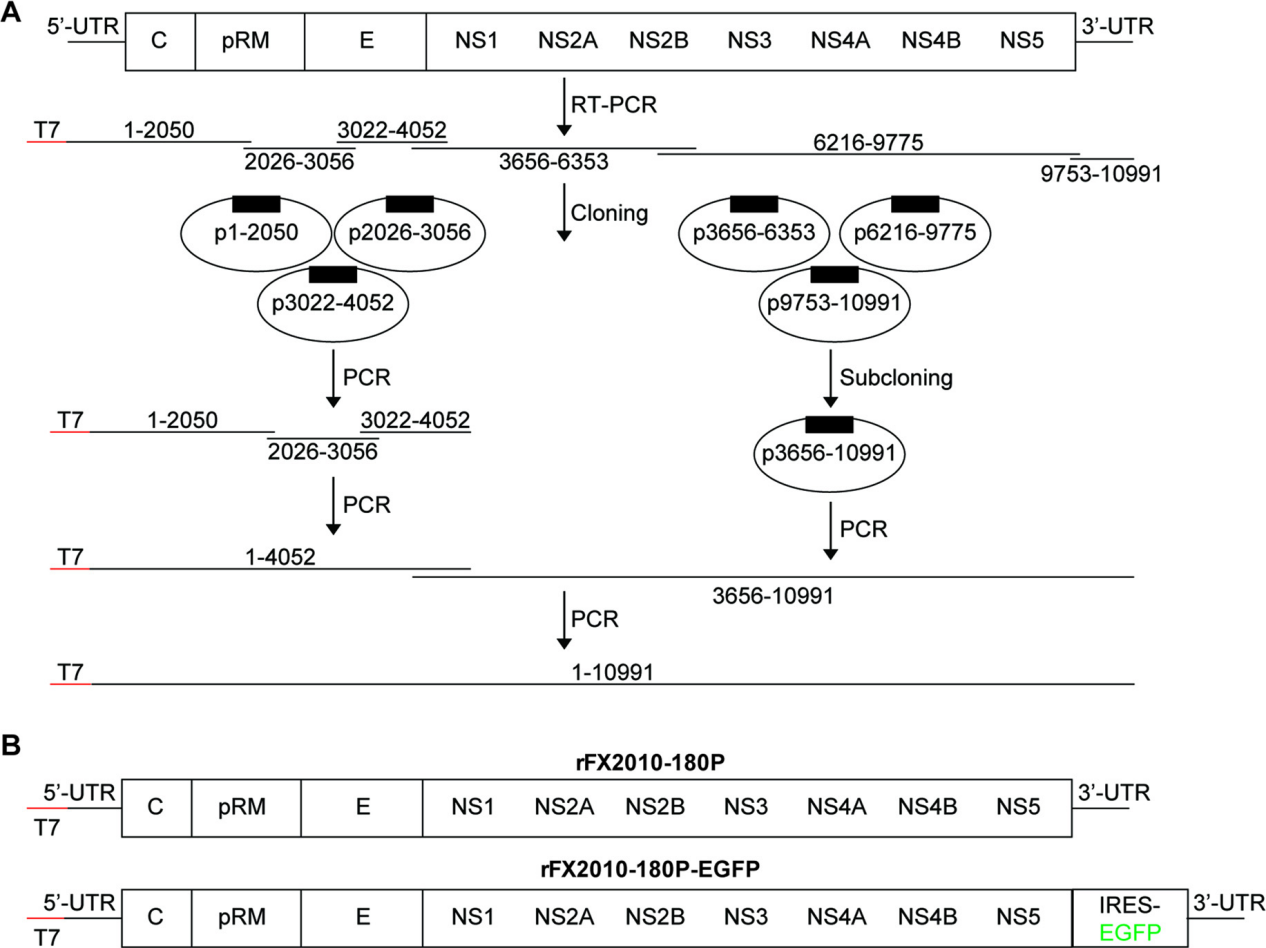
DTMUV reverse genetics strategy. (A) Cloning design. Total RNA was extracted from the DTMUV FX2010-180P strain and reverse-transcribed into cDNA. Six cDNA segments were amplified using High Fidelity DNA polymerase *pfx* and inserted into plasmid vectors. The three 3’ cDNA segments were sub-cloned into plasmid p3656-10991. To generate the full-length DNA corresponding to the entire viral genome, a series of PCRs was performed using using *pfu* Ultra II Fusion HS DNA polymerase with the recombinant plasmids as templates. (B) Genome schematics of rFX2010-180P and rFX2010-180P-EGFP. rFX2010-180P-EGFP expresses eGFP from an IRES located between the NS5 gene and the 3’ UTR.

The cloning strategy is illustrated in **Fig 1**. Total RNA was extracted from FX2010-180P strain using RNAiso regent (Takara, Japan), and reverse transcription was performed to synthesize first strand cDNA by using specific primers (**Table 1**) and the SuperScript III Reverse Transcription Kit (Invitrogen, USA). Six cDNA segments of FX2010-180P were amplified using High Fidelity DNA polymerase *pfx* (Invitrogen, USA) and the first strand cDNA as template, purified using an Agarose gel DNA Extraction Kit (Axygen, USA), phosphorylated using the T4 Polynucleotide Kinase Kit (Takara, Dalian), and then cloned into the pSIMPLE18 EcoRV BAP vector (Takara, Dalian). The T7 RNA polymerase promoter was introduced into the first segment using specific primers. The inserted sequences containing nucleotides (nts) 6,216–9,775 and 9,753–10,991 were then sub-cloned into the plasmid containing nts 3,656–6,353, in which AgeI and NotI restriction sites were introduced at the 3’ end. The target DNA fragments 6216–9775 were digested with EcoRV at nts 6,227–6,232 and with AgeI at nts 9,762–9,767, and then linked with plasmids containing nts 3,656–6,353 digested with the same enzymes. The target DNA fragment nts 9,753–10,991 was inserted into the resultant plasmid between the AgeI and NotI sites. The plasmid containing nts 3,656–10,991 was designated p3656-10991.

**Table 1:** Primers used in this study.

Application	Primer name	Sequence
Reverse transcription	FXART1	ACACATCGGGTAGGTC
Reverse transcription	FXART2	CCTTCTCAAAATCTCC
Reverse transcription	FXART3	ATCTCTGTGATGCTCC
Reverse transcription	FXART4	GTCAACCTTGTCCCGC
Reverse transcription	FXART5	TGCCGTCGCTGGTCTCAA
Reverse transcription	FXART6	TAACCTCTCACTTCCT
Reverse transcription	FXART7	AGACTCTGTGTTCTAC
PCR amplification	FXA-1F	CCCGGGTAATACGACTCACTATAGGGAGAAGTTCATCTGTGTGAACTTATTCC
PCR amplification	FXA-2050R	GAGGTCGACACGTATGGGTTGACTGTTATC
PCR amplification	FXA-2026F	CAGTCAACCCATACGTGTCGACCTCCTCCA
PCR amplification	FXA-3056F	GCTCAGGTCACTGTGCACAGCTCCATTTCC
PCR amplification	FXA-3022F	TTAAAGGAAATGGAGCTGTGCACAGTGACC
PCR amplification	FXA-4052R	GATTCCCAGGGTTAGCACACCTATTCTCAC
PCR amplification	FXA-3656F	AGGAATCACGTACAGTGATCTGGTC
PCR amplification	FXA- 6353R	AAATATGCGGCCGCCACAACCGGTCTCACCTGACTTTGTCCATAACTCC
PCR amplification	FXA-6216F	CTCCCTGTCTGGATATCGTACAAGG
PCR amplification	FXA-9775R	TTTCTCCAACCGGTTGAGGGCT
PCR amplification	FXA-9753F	AAGCCCTCAACCGGTTGGAGAAACT
PCR amplification	FXA-10991R	AAATATGCGGCCGCAGACTCTGTGTTCTACCACCACCAG
PCR amplification	FXA-10991R	AGACTCTGTGTTCTACCACCACCAGCCACACTTTC
Sequencing	FXS-1F	AGAAGTTCATCTGTGTGAACTTATTCC
Sequencing	FXS-1512R	CGGTACCATAATCCTCCATCTCAGC
Sequencing	FXS-1397F	GGAAGCGAGCACCTACCACAAT
Sequencing	FXS-2892R	CTGGGCACTCTTTAGTTTTTGGTCC
Sequencing	FXS-2784F	GGAGAGCTCATGTACGGATGGAAGA
Sequencing	FXS-4018R	TCTATCCCCACTATTCTGAGCCCTG
Sequencing	FXS-3834F	CTTGGCGTTGCGTTAGCACTCAT
Sequencing	FXS-5302R	CCTTTCAGTGCTTCCGCTATTTCAG
Sequencing	FXS-5145F	AAAAGGCAACTAACAGTGCTGGACC
Sequencing	FXS-6749R	GGCTGGGACTTCTGCTATCCATAAC
Sequencing	FXS-6537F	TGACTACAGCTGAGAAAGGGAGCAG
Sequencing	FXS-8088R	AGCAGTGTGTCAGATGGTTCAGTCG
Sequencing	FXS-7955F	GTGAGAGGTTACACAAAAGGAGGGC
Sequencing	FXS-8994R	CTTGCAGGTGCAGTTCTCTCTCTCT
Sequencing	FXS-8862F	GAGAAGGTGAATAGTAACGCAGCCC
Sequencing	IRESS-1F	GCCCCTCTCCCTCCCCCCCCCCTAA
Sequencing	EGFPS-1305R	CTTGTACAGCTCGTCCATGCCGAGA
Sequencing	FXS-10991R	AGACTCTGTGTTCTACCACCACCAG

### Insertion of IRES and eGFP gene between NS5 gene and the 3' UTR

An internal ribosomal entry site (IRES) and the eGFP gene were amplified from the pIRES-eGFP plasmid and inserted between the NS5 gene and the 3' untranslated region (UTR) of FX2010-180P by using fusion PCR. The final PCR product was digested with NotI and ApalI, ligated into p3656-10991, and designated as p3656-EGFP-10991.

### Production of the full-length FX2010-180P genome

To generate a full-length DNA corresponding to entire FX2010-180P genome, a series of PCRs was performed using *pfu* Ultra II Fusion HS DNA polymerase (Agilent, USA) with appropriate plasmid templates. Four overlapping cDNA segments of FX2010-180P were amplified from four recombinant plasmids p1-2050, p2026-3056, p3022-4052 and p3656-10991 as templates by using PCR. The T7 RNA polymerase promoter was introduced into the 5’ end of the cDNA fragment of nts 1–2050. Then the cDNA fragment of nts (T7)-1-4052 were amplified by PCR using the three overlapped cDNAs (T7)-1-2050, 2026–3056 and 3022–4052 as templates. Finally, the full-length cDNA was produced by PCR using the cDNA of (T7)-1-4052 and 3656–10991 by fusion PCR. Similar methods were used to generate an full-length cDNA of FX2010-180P genome with the IRES and eGFP gene inserted between the NS5 gene and the 3’ UTR.

### Virus rescue

Viral RNAs of FX2010-180P or FX2010-180P-EGFP were transcribed in vitro using the mMESSAGE mMACHINE T7 Kit (Ambion, USA). The transcribed RNAs were purified by using Lithium Chloride precipitation. DF-1 cells at 50–60% confluency on 6-well plate, were transfected with 5 μg of each of the purified viral RNAs using Lipofectamine LTX & Plus Reagent (Invitrogen, USA) and incubated at 37°C. At 6 hours post-transfection, the transfection medium was discarded and new DMEM with 2% fetal bovine serum was added into the wells after washing 3 times with PBS. When cytopathic effects (CPE) appeared in the transfected cells, the supernatants were collected and used to amplify the rescued viruses in DF-1 cells. The amplified virus was aliquoted and stored at -80°C for future use.

### Indirect Immunofluorescence assays

Indirect immunofluorescence assays with DTMUV and DF-1 cells have been described previously [36]. DF-1 cells on 6-well plates were incubated with 10^3.5^ TCID_50_ DTMUVs in 1 mL of phosphate-buffered saline (PBS) for 1 h at 37°C. Cells were washed with PBS to remove external viral particles and then incubated in fresh medium. At 48 h post-infection, the cells were fixed in 4% paraformaldehyde for 20 min, washed three times with PBS, blocked with 10% BSA for 20 min, and then incubated with the monoclonal antibody 1F5 for 30 min at room temperature. Cells were then incubated with fluorescein isothiocyanate-labeled goat anti-mouse antibody immunoglobulin G (IgG, 1:200 dilution; Sigma, USA), for 30 min at 37°C, washed, and mounted with 10 mM PPD (p-phenylenediamine) in glycerol:PBS (9:1), pH 8.5. Samples were observed using a fluorescence microscope.

### Genome sequencing

Viral RNA was extracted using the RNeasy Plus Mini Kit (Qiagen). Specific primers (**Table 1**) were used for RT-PCR to generate nine overlapping PCR products. The PCR products were purified using the TIANquick Midi Purification Kit (Tiangen, Beijing, China) and then sequenced on ABI 3730 automated sequencers (Applied Biosystems). Genome sequences were analyzed using DNASTAR software.

### Virus titration

Virus titers of rFX2010-180P amplified once on DF-1 cells, as well as the parental virus FX2010-180P were determined by endpoint titrations in monolayer of DF-1 cells. Cells growing on 96-well plates were infected with 10-fold serial dilutions of each sample in PBS. After 120-h incubation at 37°C, the presence of viruses was detected by assaying for cytopathic effects (CPE) using microscopy. Virus titers was calculated by using the Reed-Muench method [37].

### Viral growth kinetics

DF-1 cells were infected in triplicate in T25 flasks with viruses at multiplicities of infection of 0.0001. After incubation at 37°C for 2 h, the viral inocula were replaced with DMEM with 2% FBS. Cell culture supernatants were harvested every 12 hours and virus titers were determined as described above.

### Duck experiments

To compare the infectivity and immunogenicity of the rescued virus to the wild-type virus, groups of six 8-week-old shelducks were inoculated intramuscularly with 100 μl of either 10^5.5^ TCID50 of R-FX2010-180P or FX2010-180P. Negative control shelducks were inoculated with DMEM containing 2% FBS. Ducks were observed twice daily for signs of disease. Three ducks in each group were euthanized with CO_2_ at 4 days post inoculation (dpi), and spleen, lung, kidney, brain and ovary samples were collected for viral titration and DTMUV RNA detection. Serum samples from the remaining ducks were collected at 4, 5, 6, and 7 dpi. Those ducks were also observed twice daily until they were euthanized with CO_2_ at 14 dpi. All animal experiments were carried out in accordance with the recommendations in the Guide for the Care and Use of Laboratory Animals of the Ministry of Science and Technology of the People’s Republic of China. The protocol was approved by the Animal Care Committee of the Shanghai Veterinary Research Institute.

### Real-time PCR

To quantify DTMUV RNA copies in the homogenized/clarified organ tissue samples, a real-time RT-PCR assay with minor modification was performed as previously described [38, 39]. Tissues were homogenized in PBS to yield 1:1 (ml/g of duck tissue or ml/organ of mouse tissue) tissue homogenates, and clarified by centrifugation at 12,000 rpm for 10 min at 4°C. Supernatants (300 μl) were used for DTMUV RNA quantification. Total RNAs from sera or the homogenized/clarified organ tissue samples were extracted using the RNeasy Plus Mini Kit (Qiagen) and reverse transcription was performed using M-MLV reverse transcriptase (Takara Biotechnology, Dalian, China). Real-time PCR was performed on a Mastercycler ep realplex system (Eppendorf, Hamburg, Germany) using cycling conditions described previously [38, 39]. The RNA copies in the sample were calculated by comparing with previously-characterized standards [38, 39].

### Blocking ELISA

Blocking ELISAs were performed as previously described [36]. ELISA plates were coated with purified DTMUV antigen in 0.1 M carbonate–bicarbonate buffer (pH 9.6) and incubated overnight at 4°C. Antigen-coated plates were washed with PBS (pH 7.4) containing 0.05% Tween-20 (PBST), and blocked with PBS containing 5% skim milk for 1 h at 37°C. Serum samples were initially diluted 10-fold with PBS, and then further diluted through a series of 2-fold dilutions. Diluted serum (100 μl) was added to each well and incubated for 1 h at 37°C. The wells were washed 3 times with PBST and incubated with mAb 1F5 for 1 h at 37°C. After rinsing in PBST, goat anti-mouse IgG (Sigma, USA) conjugated to HRP was added, and the mixture was incubated at room temperature (RT) for 1 h. After rinsing in PBST, 100 μl of 3,3’, 5,5’-tetramethyl benzidine was added, and incubated at RT for 5 min. The reaction was stopped by adding 0.1 N sulfuric acid. The optical density (OD) was measured at 450 nm, and the percent inhibition (PI) value was determined using the formula: PI (%) = [1-(OD450 nm of test serum/OD450 nm of negative control serum) * 100%. The serum was considered positive for DTMUV reactivity when the PI value was ≥ 18.4%. When the PI value was ≤ 12.6%, the serum was considered negative. Intermediate PI values were considered as“borderline positive”. Repeated analyses were performed on sera with borderline PI values and were considered negative when the re-tested values were less than 18.4%. The highest dilution of the serum considered positive to DTMUV was determined as the blocking ELISA titer for the anti-DTMUV antibody.

### Genetic stability of rFX2010-180P-EGFP on DF-1 cells

To test the stability of recombinant virus on DF-1 cells, rFX2010-180P-EGFP was passed serially on DF-1 cells. When the cells reached 80–90% confluence, they were washed with PBS and infected with FX2010-180P-EGFP at a multiplicity of infection (MOI) between 0.1 and 0.5. After 1.5 h incubation at 37°C, the inoculum was removed. The cells were washed 3 times with PBS and 10 mL of DMEM containing 2% (v/v) FBS was added to the culture and incubated at 37°C, 5% CO_2_. Cells were observed under a fluorescence microscope and culture medium was harvested at 72 hours post-infection. Cell debris was removed by centrifugation, and the virus was stored at −70°C. The same procedure was used to study 3 additional viral passages. The harvested culture from each passage was used for genome sequencing.

### Prediction of RNA structure

To predict the RNA secondary structure changes between rFX2010-180P and the degenerated rFX2010-180P-EGFP virus, 840 nts and 854 nts at the 3’terminal of genomes of these two viruses were analyzed using RNAstructure 5.3 software.

## Results

### Synthesis of the full-length PCR products

Four recombinant plasmids that contained one of four overlapped cDNA segments that covered the entire DTMUV genome were generated and designated as p1-2050, p2026-3056, p3022-4052, and p3656-10991. The full-length cDNA were generated and the T7 promoter sequence was introduced at the 5’ terminus successfully (**Fig 1A**). The full-length cDNA of FX2010-180P containing IRES and the eGFP gene between NS5 gene and 3’ NCS was produced using similar procedures. The final constructs were designed as FX2010-180P and FX2010-180P-EGFP (**Fig 1B**).

### Virus Rescue

**Fig 2:**
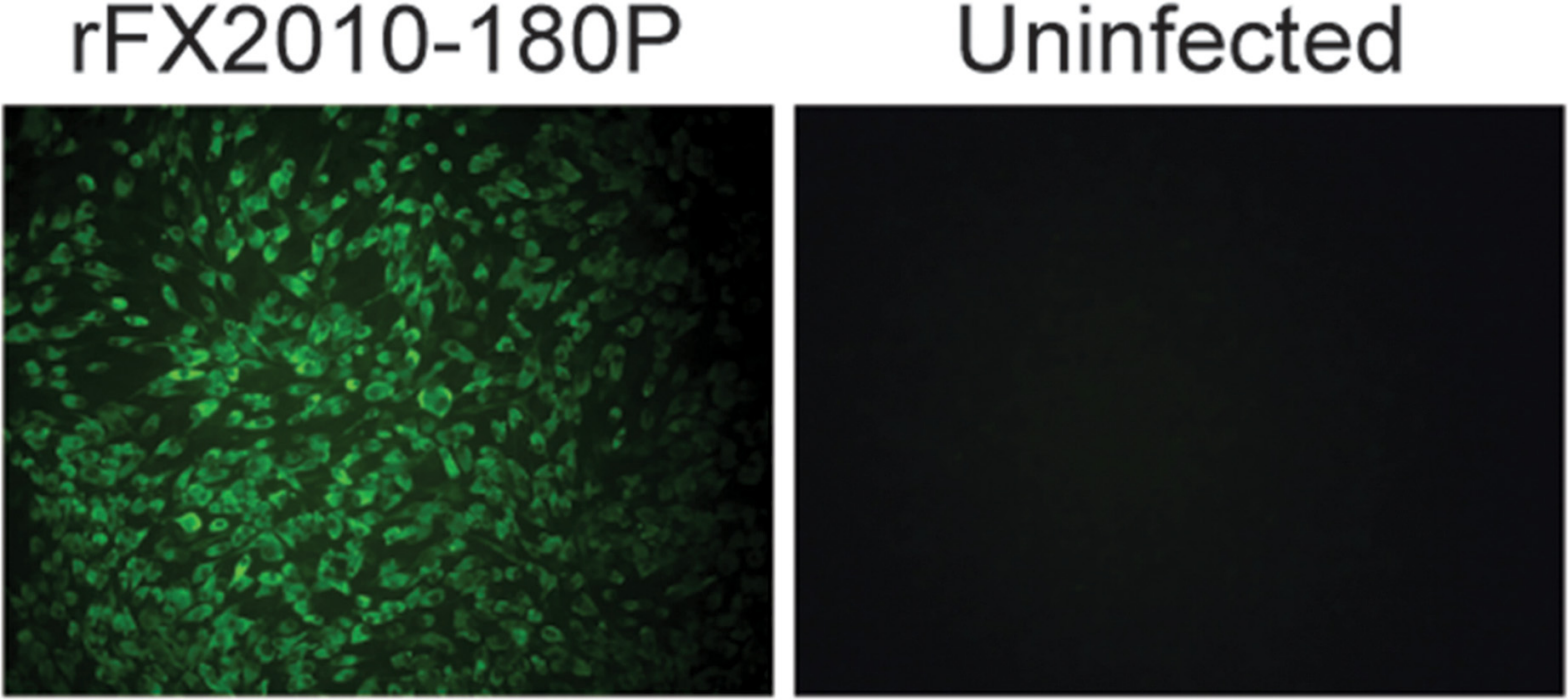
Indirect immunofluorescence microscopy of DF-1 cells infected with the rescued rFX2010-180P virus as detected using the monoclonal antibody 1F5. (A) rFX2010-180P-infected cells. (B) Uninfected cells.

Viral RNAs of FX2010-180P or FX2010-180P-EGFP were transcribed in vitro and then transfected into DF-1 cells. CPE on the viral RNA transfected cells appeared at 48 hours post-transfection, while no CPE was found in non-transfected cells. Transfected cell supernatants were harvested and then used for amplification on DF-1 cells. DF-1 cells infected with the rescued rFX2010-180P virus were analyzed using indirect immunofluorescence microscopy with the monoclonal antibody 1F5. Cells infected with the virus showed obvious green fluorescence at 48 hours post-infection, while no fluorescence was detectable in uninfected cells (**Fig 2**).

To determine whether the rescued virus produced using reverse genetics differed from the wild-type parental virus, rFX2010-180P was passaged once on DF-1 cells and then subjected to whole genome sequencing. No mutations were found in comparison with the parental virus sequence (data not shown).

### Growth kinetics on DF-1 cells

**Fig 3:**
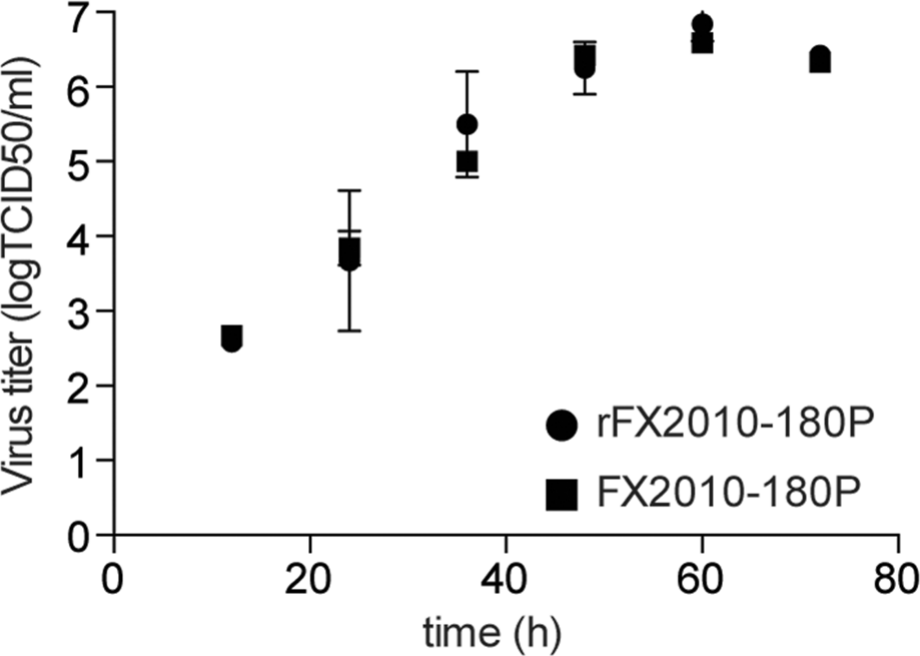
Viral growth rates. DF-1 cells were infected with rFX2010-180P or FX2010-180P at multiplicities of infection of 0.1 and the supernatants were titrated on DF-1 cells at the indicated time points.

To determine the growth rates of rFX2010-180P and FX2010-180P, DF-1 cells were infected at multiplicities of infection of 0.0001 (diluted from original viral stocks stored at 10^6.5^ TCID_50_/ml), and the supernatants were collected every 12 hours after infection. The rescued virus grew similarly to the parental virus (**Fig 3**).

### Virus distribution and immune responses in ducks inoculated with rFX2010-180P or FX2010-180P

**Fig 4:**
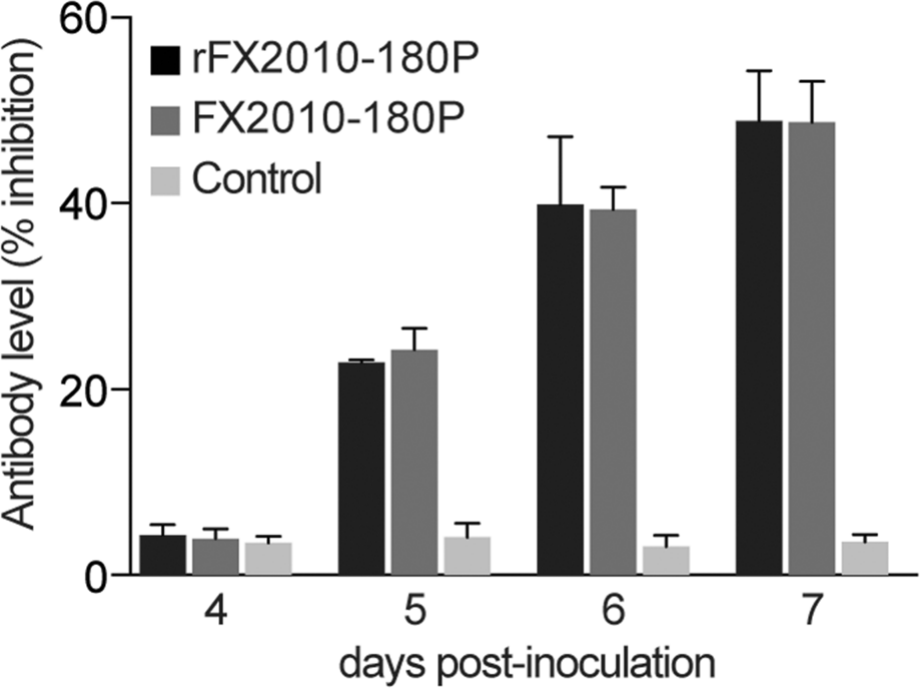
Viral antigenicity. Six 8-week-old shelducks were inoculated with 10^5.5^ TCID_50_ of rFX2010-180P or FX2010-180P intramuscularly. Antibodies generated against DTMUV in infected ducks were quantified by performing blocking ELISAs using serum obtained at 4–7 dpi.

To compare the replication and antigenicity of rescued virus and its parental virus in ducks, six 8-week-old shelducks were inoculated intramuscularly with 10^5.5^ TCID_50_ of rFX2010-180P or FX2010-180P, respectively. Viral RNA copies in different tissue samples were quantified by real-time PCR at 4 dpi. All the tissues tested were negative for DTMUV RNA, except for the spleens. In spleen samples, the virus titers did not differ between rFX2010-180P and FX2010-180P (4.98 ± 0.85 log(copies/g) vs. 5.07 ± 0.57 log(copies/g)), respectively. To determine whether the rescued virus could induce antibodies against DTMUV at similar titers as compared with the parental virus, sera samples were collected at 4, 5, 6 and 7 dpi and tested by blocking ELISAs. Antibody titers started to increase at 5 dpi and reached high levels at 7 dpi in both groups. The rescued virus and the parental virus induced similar levels of antibody (**Fig 4**) while sera of uninfected ducks were negative. None of animals showed any clinic signs and all survived until the end of the experiment.

### Rescue of rFX2010-180P-EGFP expressing exogenous eGFP protein

**Fig 5:**
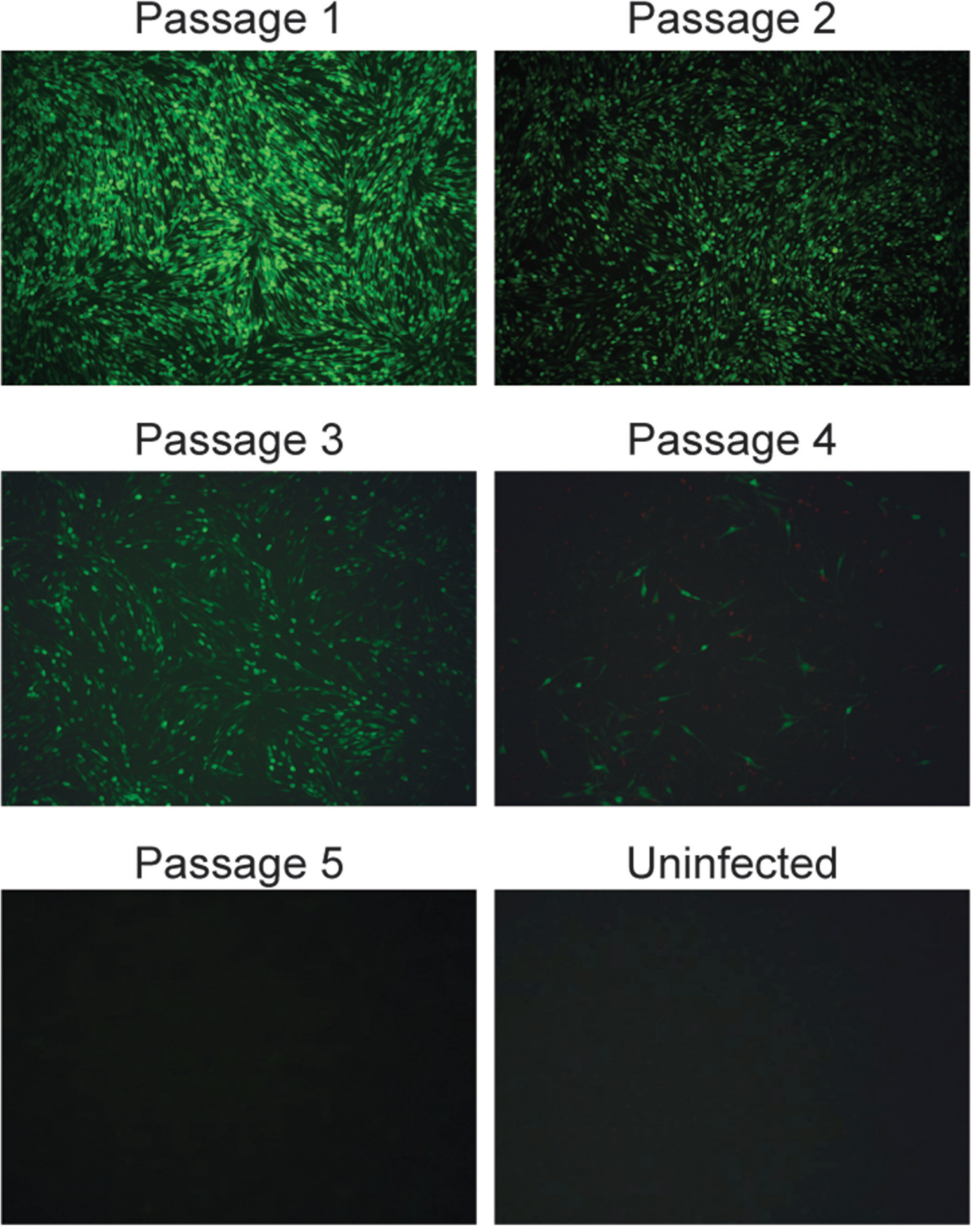
GFP expression from DTMUV. DF-1 cells were infected with rFX2010-180P-EGFP and observed using fluorescence microscope. Fluorescence is shown as a function of viral passage number and in comparison with uninfected cells.

To determine whether exogenous proteins could be expressed from the rescued DTMUV, an IRES) and eGFP genes were introduced between the NS5 gene and the 3' non-coding sequence of rFX2010-180P to create rFX2010-180P-EGFP. The rFX2010-180P-EGFP was successfully rescued and expressed GFP (**Fig 5**). Serial passages of rFX2010-180P-EGFP resulted in the progressive loss of GFP expression after passage 2 (**Fig 5**), although CPEs were still obvious (data not shown).

### Stablity of degenerated rFX2010-180P-EGFP

**Fig 6:**
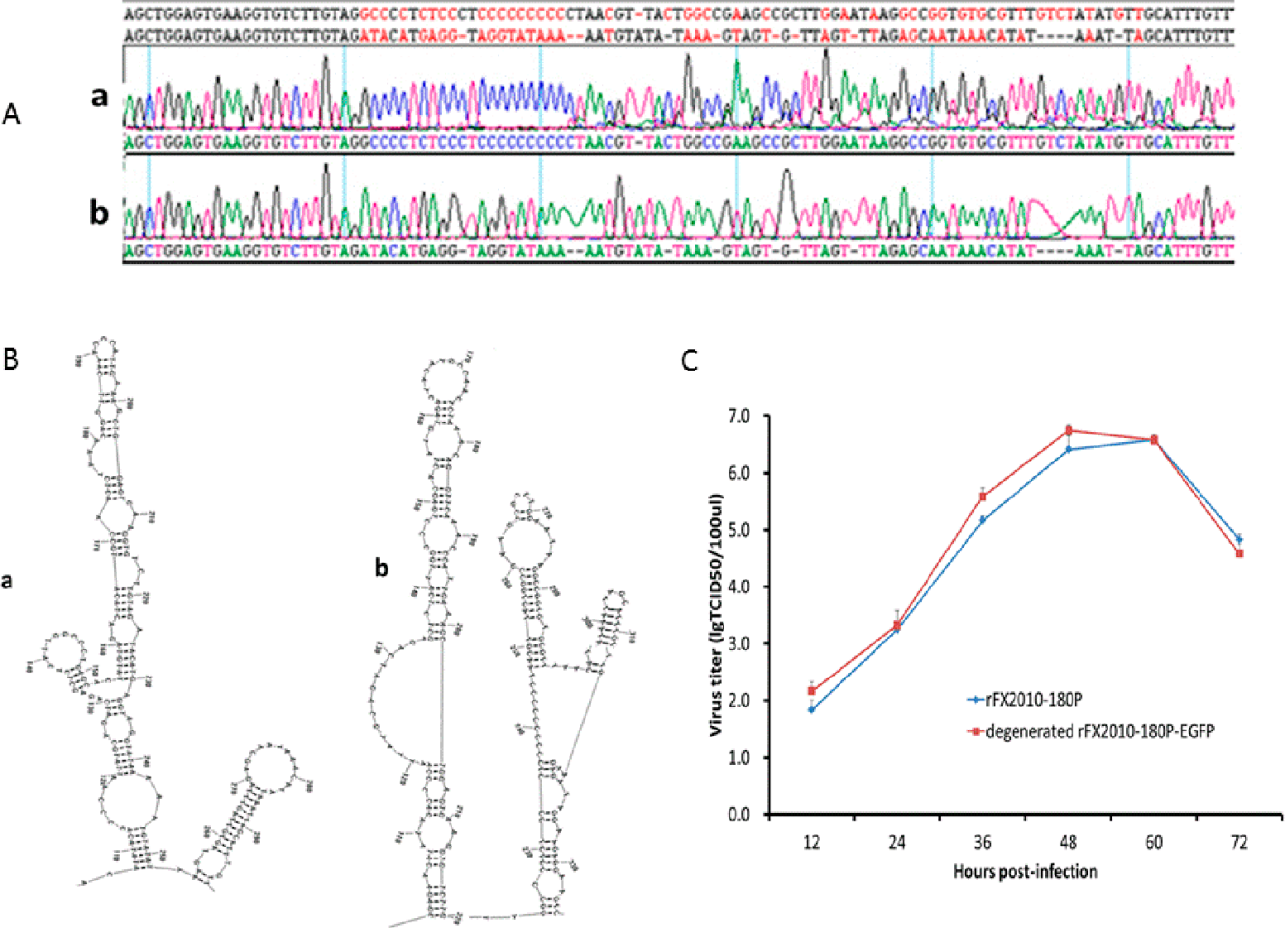
Comparison of rFX2010-180P and degenerated rFX2010-180P-EGFP. (A) Sequence differences of degenerated rFX2010-180P-EGFP (a) and rFX2010-180P (b). (B) RNA structure prediction of r-FX2010-180P (a) and degenerated rFX2010-180P-EGFP (b) using RNAstructure 5.3 software. (C) Growth kinetics of rFX2010-180P and degenerated rFX2010-180P-EGFP were compared in DF-1 cells.

Genome sequencing indicated that the loss of GFP expression was due to a deletion of 1,294 nts encompassing 1,240 nts of inserted sequence and 54 nts of the viral 3’ UTR. This resulted in the maintenance of 68 exogenous nts between the NS5 gene and the 3’ UTR. When the viral sequences from different passages were compared, the deletion was detectable after the first passage and became predominant after third passage. The 54 nts deleted virus with 68 exogenous nts was exclusive and no other deletions were detected in the fifth passage, which suggested that the virus was stable in DF-1 cells. Sequence analysis showed that the deleted viral 54 nts had low identity to the 68 exogenous nts (**Fig 6A**). The RNA structures of r-FX2010-180P and degenerated rFX2010-180P-EGFP were predicted using RNAstructure 5.3 software. This analysis suggested that both the deleted 54 nts and the inserted 68 nts formed two adjacent stem-loops with nearby nucleotides (**Fig 6B**). The growth kinetics of rFX2010-180P and degenerated rFX2010-180P-EGFP were compared in DF-1 cells and no significant difference was found between these two viruses (P>0.05) (**Fig 6C**). The fact that 68 exogenous nts were maintained between the NS5 gene and the 3’ UTR leaves open the possibility that this region of the genome might be useful for future experiments that seek to insert foreign genes into DTMUV.

## Discussion

We reported the development of a high-fidelity PCR-based reverse genetics system in an attenuated DTMUV strain. In this system, the genome of the rescued virus had no mutations as compared with the parent viral sequence. This is an improvement upon a previous study in which rescued DTMUV clones from cDNAs derived from a JXSP-AN contained multiple mutations [25]. Although this rescued virus behaved similarly to the parental virus in infection experiments, the large number of mutations limited its utility in future studies. It was reported that an infectious full-length DTMUV cDNA clone was extensively mutated in bacterial hosts [25]. In our study, the plasmid containing nts 2,000–4,000 of the FX2010-180P genome was found to be unstable in *E*. *coli* hosts. The possible mechanism of instability of plasmids containing FX2010-180P genome might be due to the potential presence of uncharacterized promoters or to the potential production of peptides that are toxic to *E*. *coli*. Here we developed a system in which the viral genome was more extensively fragmented and four stable recombinant plasmids containing p1-2050, p2026-3056, p3022-4052 and p3656-10991 were constructed separately. This resulted in stable maintenance of the viral gene sequences in *E*. *coli* and facilitated downstream amplification and synthesis of the full-length genome. The rescued virus did not contain any unexpected mutations, suggesting that the PCR-based reverse genetics system established in this study was more reliable than the full-length DTMUV cDNA clone [25]. In addition, the plasmids used in our PCR-based reverse genetics system were easy to construct and can be used for future studies of gene substitutions or point mutations.

The extent to which foreign genes can be inserted into flavivirus genomes using reverse genetics is also of interest. Yellow fever vaccine 17D had been used successfully as a vector for expressing the Lassa virus glycoprotein precursor or glycoprotein subunits, as well as the simian immunodeficiency virus SIVmac239 Gag sequences [40, 41]. Here we developed methods by which to express eGFP from an IRES located between the NS5 gene and the 3’ UTR. We observed robust GFP expression initially, with progressive loss of fluorescence upon viral passage. However, 68 foreign nts were maintained, suggesting that the region between NS5 and the 3’ UTR could be a possible target for future viral engineering. Overall, the establishment of the DTMUV reverse genetics system provides a foundation for further studies of the molecular basis of the pathogenicity of Tembusu virus and for the development of novel vaccines.
